# Icariin alleviates osteoarthritis by inhibiting NLRP3-mediated pyroptosis

**DOI:** 10.1186/s13018-019-1307-6

**Published:** 2019-09-11

**Authors:** Yan Zu, Yue Mu, Qiang Li, Shu-Ting Zhang, Hong-Juan Yan

**Affiliations:** 1 School of Mechanical and Material Engineering, North China University of Technology, No.5, Jinyuanzhuang Road, Shijingshan District, Beijing, 100144 People’s Republic of China; 2 Department of Stomatology, Peking Union Medical College Hospital, Chinese Academy of Medical Sciences, Beijing, 100730 People’s Republic of China

**Keywords:** Icariin, Osteoarthritis, NLRP3 inflammasome, Caspase-1 signaling, Pyroptosis

## Abstract

**Background:**

Osteoarthritis (OA) is the common chronic degenerative joint bone disease that is mainly featured by joint stiffness and cartilage degradation. Icariin (ICA), an extract from *Epimedium*, has been preliminarily proven to show anti-osteoporotic and anti-inflammatory effects in OA. However, the underlying mechanisms of ICA on chondrocytes need to be elucidated.

**Methods:**

LPS-treated chondrocytes and monosodium iodoacetate (MIA)-treated Wistar rats were used as models of OA in vitro and in vivo, respectively. LDH and MTT assays were performed to detect cytotoxicity and cell viability. The expression levels of NLRP3, IL-1β, IL-18, MMP-1, MMP-13, and collagen II were detected by qRT-PCR and Western blotting. The release levels of IL-1β and IL-18 were detected by ELISA assay. Caspase-1 activity was assessed by flow cytometry. Immunofluorescence and immunohistochemistry were used to examine the level of NLRP3 in chondrocytes and rat cartilage, respectively. The progression of OA was monitored with hematoxylin-eosin (H&E) staining and safranin O/fast green staining.

**Results:**

ICA could suppress LPS-induced inflammation and reduction of collagen formation in chondrocytes. Furthermore, ICA could inhibit NLRP3 inflammasome-mediated caspase-1 signaling pathway to alleviate pyroptosis induced by LPS. Overexpression of NLRP3 reversed the above changes caused by ICA. It was further confirmed in the rat OA model that ICA alleviated OA by inhibiting NLRP3-mediated pyroptosis.

**Conclusion:**

ICA inhibited OA via repressing NLRP3/caspase-1 signaling-mediated pyroptosis in models of OA in vitro and in vivo, suggesting that ICA might be a promising compound in the treatment of OA.

**Electronic supplementary material:**

The online version of this article (10.1186/s13018-019-1307-6) contains supplementary material, which is available to authorized users.

## Introduction

Osteoarthritis (OA) is a long-term joint bone disorder which is recognized by the progressive damage of joint structure, such as articular cartilage and subchondral bone [1]. OA is now considered as a significant clinical problem worldwide as the increase of aging populations. Currently, the treatment agents have failed to block the progression of OA, thus safer and better-tolerated drugs for OA therapy are needed [2]. During the progression of OA, pathological events occur in the cartilage including oxidative stress, inflammation, apoptosis, loss of cartilage matrix, and autophagy. The studies of genetic mouse models demonstrated that growth factors, such as transforming growth factor-β (TGF-β), Wnt3a, and Indian hedgehog, as well as signaling molecules, such as β-catenin, Smad3, and HIF-2α, are involved in OA progression [3–5]. Inflammation and extracellular matrix (ECM) loss are increasingly recognized as essential drivers of OA cartilage injury [6, 7]. Understanding the regulation of these changes may provide effective therapy for OA.

Pyroptosis is a caspase-1-required inflammasome-mediated programmed cell death signal pathway. Pyroptosis can lead to IL-1β and IL-18 production as well as macrophage cell lysis [8]. Distinct from apoptosis and simple cell necrosis, the process of pyroptosis is proinflammatory and mediated by nod-like receptor protein-3 (NLRP3) inflammasome and caspase-1 signaling. A recent study demonstrated that NLRP inflammasomes NLRP1 and NLRP3 participated in the mediation of lipopolysaccharide (LPS)-induced fibroblast-like synoviocytes (FLSs) pyroptosis [9]. The inhibition of these two NLRP inflammasomes led to a significant decline of pyroptosis-related cytokines, suggesting NLRP1 and NLRP3 inflammasomes might play an important role in the pathogenesis of OA. One study presented that hydroxyapatite crystals of a particular size and shape were able to promote the secretion of proinflammatory cytokines IL-1β and IL-18 from murine macrophages, which was dependent of NLRP3 inflammasome [10]. And the level of NLRP3, IL-1β, and IL-18 is increased in the synovial membrane of knee osteoarthritis rats, indicating the involvement of inflammasome in OA [11]. However, the role and underlying mechanism of NLRP3-mediated pyroptosis in OA is still unknown.

The Chinese traditional medical herb *Epimedium* had long been used to attenuate the OA process. *Epimedium* could significantly moderate OA condition and decline the expression level of urokinase plasminogen activator (uPA), uPA receptor (uPAR), and urokinase plasminogen activator inhibitor (PAI) in rabbit OA model [12]. Icariin (ICA) which is the extract of *Epimedium* was identified as the effective constituent, showing anti-osteoporotic and anti-inflammatory effects [12]. Studies revealed that ICA prevented OA inflammation and chondrocytes apoptosis though activation of autophagy via inhibiting NF-κB signaling pathway [13]. Furthermore, ICA exerted a chondroprotective effect through the inhibition of MMP-1, MMP-3, and MMP-13 or the suppression of osteoprotegerin (OPG), receptor activator of nuclear factor kappa-B ligand (RANKL), and receptor activator of nuclear factor kappa-B (RANK) system via MAPK pathway in IL-1β-stimulated chondrosarcoma cells [14, 15]. However, the molecular mechanisms of ICA alleviating OA and its relationship with NLRP3 inflammasome are not fully understood.

In this study, we demonstrated that ICA reduced LPS-induced pyroptosis and the inhibition of collagen formation through suppressing NLRP3 inflammasome-mediated caspase-1 signaling pathway. The effect of ICA alleviating OA by inhibiting NLRP3-mediated pyroptosis was further confirmed in rat OA model. Thus, ICA may play therapeutic roles in OA treatment.

## Materials and methods

### Isolation and culture of rat chondrocytes

Cartilage slices were shaved from the articular surface of both knee joints from adult male Wistar rats. Pronase solution (2%, 1.5 mL) was used to treat the pooled cartilage slices for 20 min at 37 °C, and the rest of pure cartilage was rinsed with phosphate-buffered saline (PBS) for twice. Collagenase solution (1.5 mL) was then added and the samples were incubated again at 37 °C until the extracellular matrix was completely digested. The chondrocytes were then filtered through a mesh screen and the resulted single-cell suspension was centrifuged at 1500*g* for 10 min. The cells were transferred to T-25 cell culture flasks and incubated with complete Dulbecco’s modified Eagle’s medium (DMEM) at 37 °C in a 5% CO_2_ incubator.

### Cell transfection

The full-length NLRP3 sequence was constructed into pcDNA3.1, and these reconstructed plasmids were named pcDNA3.1-NLRP3. The empty plasmid was employed as the negative control. Cells were inoculated in a 6-well plate at a density of 3 × 10^5^/well. When cell confluence reached 80%, cells were transfected with different plasmids using Lipofectamine 2000 kit (Invitrogen Inc., Carlsbad, CA, USA). The target plasmids (4 μg) and lipofectamine 2000 (10 μL) were separately diluted with 250 μL serum-free Opti-MEM (Gibco Company, NY, USA). The two dilutions were allowed to stand at room temperature for 5 min and mixed evenly. The mixture was added to the culture well after being placed for 20 min, and then cultured in an incubator with 5% CO_2_ at 37 °C. The complete medium was employed for subculture after 6 h, and cells were collected 48 h afterward.

### Cell viability

Rat chondrocytes were seeded in 96-well plates (1 × 10^4^ cells/well), and these cells were treated with ICA (1, 2.5, 5, 10, 20 and 40 μM) for 24 h. Then, 20 μL of MTT (5 mg/mL in PBS) was added to each well, and the plates were incubated at 37 °C for 4 h. After removing the supernatants, 150 μL dimethylsulfoxide was supplemented to each well. The plates were then shaken for 10 min, and optical density (OD) values were detected and recorded at 570 nm using a microplate reader (Bio-Rad, CA, USA).

### Flow cytometry analysis of caspase-1 activity

To measure pyroptosis in chondrocytes, the level of active caspase-1 was detected by caspase-1 Detection Kit (ImmunoChemistry, Bloomington, MN, USA). FAM-YVAD-FMK was used to label active caspase-1 enzyme and determined by flow cytometry following the manufacturer’s instruction. Briefly, the cell suspension was incubated with FAM-YVAD-FMK for 60 min at 37 °C in the dark. After centrifugation, the supernatant was removed by aspiration and the cell pellet was washed twice with 1 × wash buffer. Cells were resuspended in PI staining buffer and kept on ice. The cells were analyzed by a flow cytometer (BD Biosciences, San Jose, CA, USA), and pyroptosis was defined as double positive for FAM-YVAD-FMK and PI staining.

### LDH release and ELISA assay

Cytotoxicity was measured by quantifying lactate dehydrogenase (LDH) release in the cell supernatant by colorimetric assay following the manufacturer’s instructions (Clontech, Mountain View, CA, USA). The levels of IL-1β and IL-18 in chondrocyte supernatants were measured using IL-1β or IL-18 enzyme-linked immunosorbent assay (ELISA) kits (R&D Systems, USA) according to the manufacturer’s instructions. The optical density (OD) of the extracted lysate supernatant was measured and recorded at 630 nm with a microplate reader.

### Immunofluorescence

The chondrocytes, cultured on cover slips for 48 h, were fixed with 4 % paraformaldehyde solution (Santa Cruz Biotechnology, USA), followed by treatment with blocking solution containing 5 % donkey serum and 0.1 % Triton (Sigma Aldrich, Germany) in PBS. Subsequently, the samples were incubated with the polyclonal anti-Rat NLRP3 antibody (1:60, abcam, USA) overnight at 4 °C followed by washing three times with PBS and incubating with the anti-mouse IgG secondary antibody (1:200, abcam, USA) and DAPI (Roche Applied Science, Germany) for 1 h at room temperature. The images were collected by an Olympus FluoView FV10i confocal microscope.

### OA rat model

Male Wistar rats were purchased from human SJA Laboratory Animal Co., Ltd (Changsha, China). All procedures were performed according to the internationally accepted ethical guidelines. Rats were housed under standard laboratory conditions with free access to food and water with room temperature at 22 °C and humidity about 45~55%. After 1 week of acclimatization, rats were divided randomly into three groups (*n* = 5 per group) as follows: (1) control group: no monosodium iodoacetate (MIA) injection; (2) OA group: MIA injection; and (3) OA + ICA group (MIA + ICA): ICA injection 2 weeks post MIA injection. According to the grouping, rats were directly injected with MIA (40 mg/mL in 0.9% NaCl solution, 50 μL) or 0.9% NaCl solution (50 μL) into the intra-articular space of the right knee except for the control group. And after 2 weeks, rats in the OA + ICA group were injected with ICA (20 μM, 0.3 mL). After 32 days, cartilage block with subchondral bone was isolated for the subsequent experiments.

### Hematoxylin-eosin (H&E) staining and safranin O/fast green staining

Cartilage block with subchondral bone was cut into 1.0 cm × 1.0 cm × 0.5 cm-sized blocks and these blocks were fixed for 3 days with neutral formalin solution, followed by decalcification for 14 days in 30% formic acid solution and dehydration with ethanol in conventionally gradient. The samples were embedded in paraffin and cut into slices at 5 μm. For hematoxylin-eosin (H&E) staining, the cartilage samples were stained with Harris alum hematoxylin (Fuzhou Maixin Biotechnology, China) for 5 min after dewaxing and hydration. Following washing for 10 s in 0.5% hydrochloric acid alcohol, the slices were stained in eosin (Fuzhou Maixin Biotechnology, China) for 40 s. After dehydration, transparency, and mounting with neutral balsam, the samples were visualized under a microscope. The nucleus of the chondrocytes appeared blue and the other tissues appeared pink.

For safranin O/fast green staining, the samples were stained with Weigert’s iron hematoxylin and then rinsed with water before placed in 0.2% fast green solution (Shanghai Sangon Biological Engineering Technology, China) for 1 min, 1% ethylic acid solution for 30 s, and 0.1% safranin O solution (Shanghai Sangon Biological Engineering Technology, China) for 15 min. The samples were then dehydrated, transparentized, and mounted with neutral balsam. The normal cartilage appeared red and the background appeared green.

### Immunohistochemistry (IHC)

Paraffin wax-embedded tissue sections of subchondral bone were used for IHC. The sections were dewaxed in xylene and dehydrated with gradient ethanol. After antigen retrieval in sodium citrate buffer, the sections were incubated with anti-rat NLRP3 antibody (Abcam, USA) overnight at 4 °C or at 37 °C. After three washes in PBS (3 min each time), the tissues were incubated with biotinylated IgG (1:200, Solarbio, China) at 37 °C for 30 min, followed by 3 times PBS rinse. Afterward, fresh diaminobenzidine (DAB) (Boster Biological Technology, China) was added for coloration for 1~2 min to visualize the antibody-antigen complexes. The slices with brown or yellow cytoplasm were considered to be positive. Five typical fields were randomly selected from each slice for observation and counted under an optical microscope (Nikon, Tokyo, Japan).

### Western blotting

The cells were lysed using ice-cold RIPA buffer containing protease inhibitor (Boster Biological Technology, China). Proteins from cartilage samples were extracted as previous reports [16, 17]. Briefly, the frozen tissues were mechanically pulverized, sonicated, and stored in cold extraction buffer (Roche) at 4 °C overnight. The supernatant after centrifugation was collected and proteoglycans (PGs) was cleared by cetylpyridinium chloride (CPC). After another centrifugation, the supernatant was treated with methanol and following centrifugation, the pellet was resuspended in 2-DE sample buffer (GE Healthcare Life Sciences, Sweden). The proteins were quantified using the bicinchoninic acid protein assay kit (BCA kit) according to the manufacturer’s instructions. An equal amount of proteins (20 μg) were separated by 10% SDS-PAGE and transferred to polyvinylidene fluoride (PVDF) membranes (Millipore, USA), which were then blocked in 5% bull serum albumin (BSA, Sigma, USA) in TBST for 2 h at room temperature. The membranes were then incubated with primary antibody at 4 °C overnight. The primary antibodies were as follows: NLRP3 (1:2000, abcam, USA), ASC (1:2000, abcam, USA), caspase-1 (1:2000, abcam, USA), GSDMD (1:5000, abcam, USA), IL-1β (1:5000, abcam, USA), IL-18 (1:5000, abcam, USA), MMP-1(1:2000, abcam, USA), MMP-13 (1:2000, abcam, USA), collagen II (1:2000, abcam, USA), and GAPDH (1:1000, abcam, USA). After washing three times with TBST, the membranes were incubated with HRP-conjugated corresponding secondary antibodies (Goat Anti-Rabbit IgG, 1:5000, abcam, USA) for 1 h at room temperature. After washing again with TBST for three times, the immunoreactive proteins were visualized with the ECL western detection kit (Thermo Fisher Scientific, USA). ImageJ software was used to quantify the density of each band.

### RNA extraction and quantitative real time-PCR (qRT-PCR)

Total RNA from chondrocytes and cartilage tissue was extracted using Trizol reagent (Thermo Fisher Scientific, USA) and reverse-transcribed to cDNA with RT Master Mix (Takara Japan). The RT-PCR was performed with SYBR Master Mix using StepOne-Plus system (ABI, USA) by denaturing at 95 °C for 30 s, annealing at 60 °C for 1 min and extending at 95 °C for 5 s. The primer sequences were as follows: NLRP3 forward (5'-GTAGGTGTGGAAGCAGGACT-3') and reverse (5'-CTTGCTGACTGAGGACCTGA-3′), IL-1β forward (5′-CAGCAGCATCTCGACAAGAG-3′) and reverse (5′-CATCATCCCACGAGTCACAG-3′), IL-18 forward (5′-ATGCCTGATATCGACCGAAC-3′) and reverse (5′-TGGCACACGTTTCTGAAAGA-3′), and GAPDH forward (5′-CAAGTTCAACGGCACAG-3nc) and reverse (5′-CCAGTAGACTC CACGACAT-3′). The gene expression was analyzed by 2^−ΔΔCt^ method using GAPDH as the internal control.

### Statistical analysis

All experiments were conducted at least three times and data from the representative experiment was shown. Data were present as mean ± standard deviation (SD). Graphpad Prism 5.0 (GraphPad Software Inc., USA) was used for statistical analysis and statistical evaluation was performed by Student’s *t* test (two-tailed) between two groups or one-way analysis of variance (ANOVA) followed by Tukey post hoc test for multiple comparison. *p* values<0.05 were considered statistically significant.

## Results

### LPS induces inflammation and reduction of collagen formation in chondrocytes

**Fig. 1:**
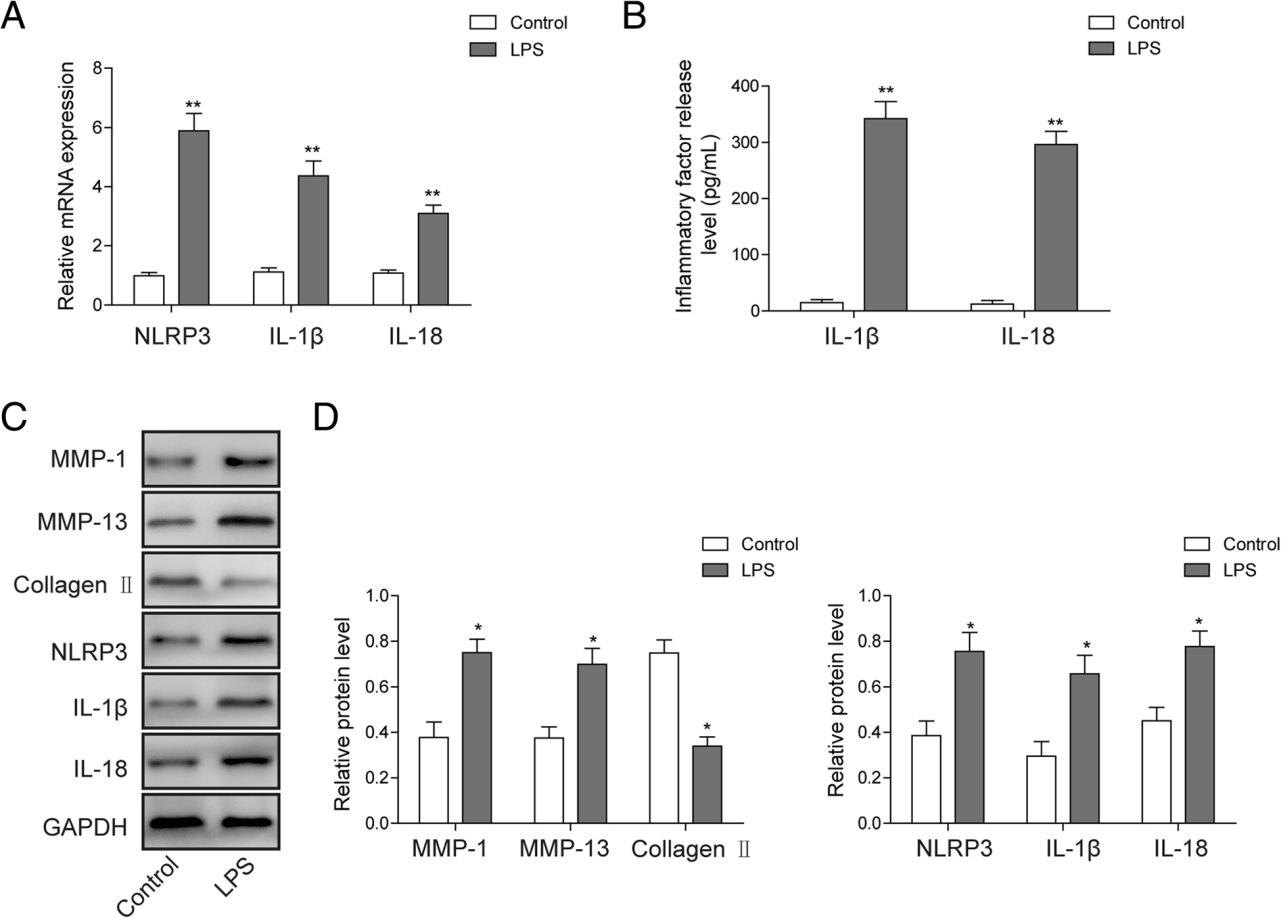
Effects of LPS on chondrocytes inflammation and collagen formation. **a** The mRNA levels of NRLP3, IL-1β, and IL-18 were analyzed by qRT-PCR in rat chondrocytes treated with LPS. **b** The release levels of IL-1β and IL-18 in the cell supernatant of LPS treated rat chondrocytes was checked by ELISA assay. **c** The expression level of collagen formation and pyroptosis-related protein was analyzed by Western blotting in rat chondrocytes treated with LPS. **d** Quantitative analysis of protein band gray in **c**. GAPDH was used as internal control. All the results were shown as mean ± SD (*n* = 3), which were three separate experiments performed in triplicate. *p < 0.05 and **p < 0.01

The expressions of NRLP3, IL-1β, and IL-18 in chondrocytes were examined by qRT-PCR following treatment of LPS. As shown in Fig. 1a, the expression of NRLP3, IL-1β, and IL-18 was dramatically induced by LPS in rat chondrocytes. In addition, the result of the ELISA assay also indicated increased release level of IL-1β and IL-18 (Fig. 1b). The expression levels of MMP-1, MMP-13, collagen II, NRLP3, IL-1β, and IL-18 in LPS-treated rat chondrocytes were monitored by Western blotting (Fig. 1c, d). The accumulation of MMP-1, MMP-13, NRLP3, IL-1β, and IL-18 proteins were induced after incubation with LPS for 12 h, while the expression of collagen II shows an opposite trend. In combination, these results indicated that LPS induces inflammation of chondrocytes and decreases collagen formation.

### ICA suppresses LPS-mediated chondrocytes injury and pyroptosis

**Fig. 2:**
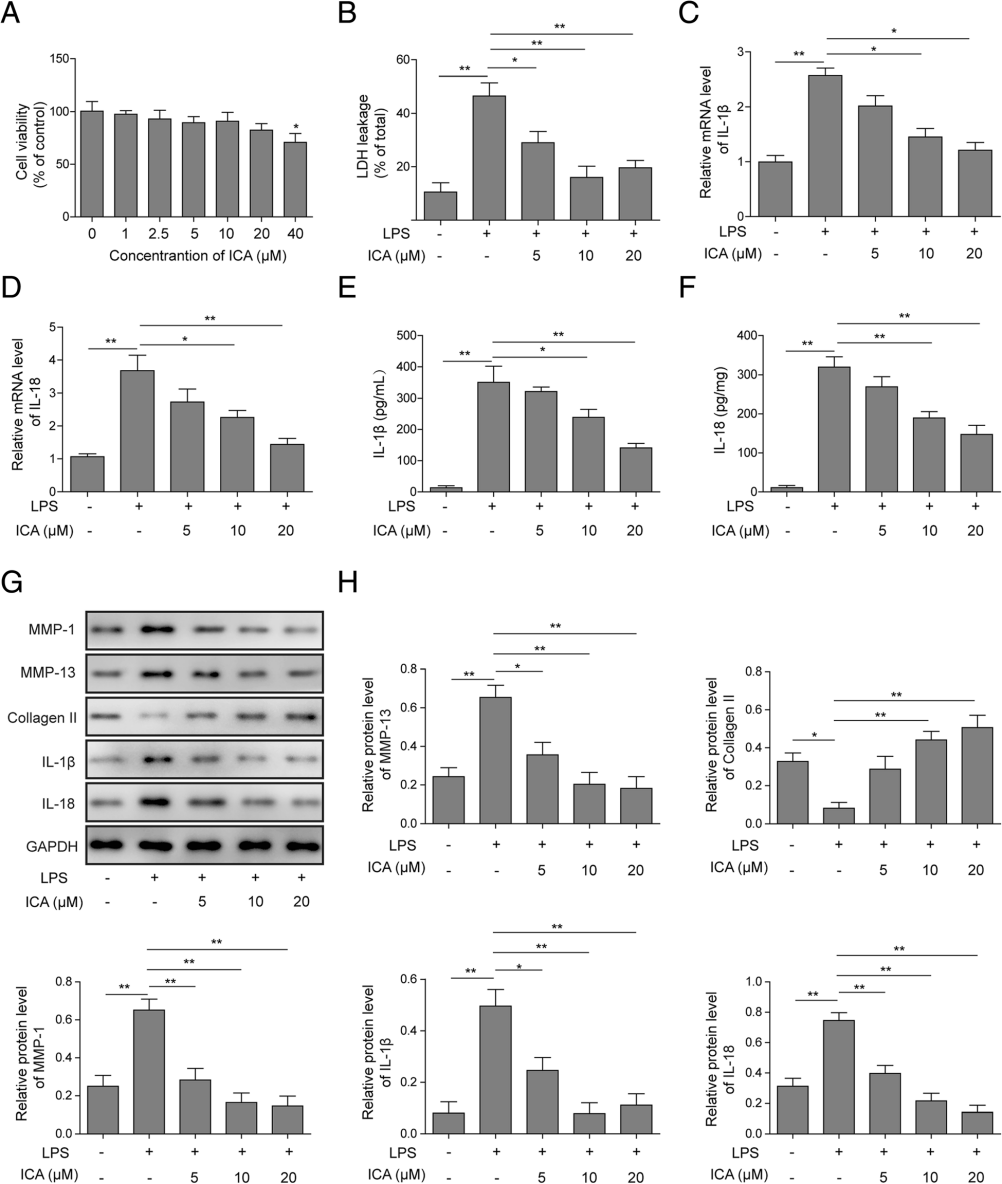
Effects of ICA on chondrocytes injury and pyroptosis. **a** The cell activity was detected by MTT assay in chondrocytes treated with various concentrations of ICA for 24 h. **b** Leakage of LDH was assessed by ELISA assay in chondrocytes treated with LPS or ICA. The mRNA levels of IL-1β (**c**) and IL-18 (**d**) were analyzed by qRT-PCR in chondrocytes treated with LPS or ICA. The release level of IL-1β (**e**) and IL-18 (**f**) were analyzed by ELISA assay in chondrocytes treated with LPS or ICA. **g** The protein level of collagen formation and pyroptosis-related protein was analyzed by Western blotting in rat chondrocytes treated with LPS or ICA. **h** Quantitative analysis of protein band gray in **g**. GAPDH was used as internal control. All the results were shown as mean ± SD (*n* = 3), which were three separate experiments performed in triplicate. *p < 0.05 and **p < 0.01

MTT assays were employed to analyze the effects of ICA on rat chondrocytes viability. ICA decreased cell viability in a concentration-dependent manner, and the application of ICA over 40 μM was able to dramatically inhibit chondrocytes viability (Fig. 2a). The lactate dehydrogenase (LDH) leakage was monitored as an indicator of pyroptosis. LPS incubation increased the extracellular LDH, while the application of ICA rescued the cells from the leakage of LDH (Fig. 2b). Moreover, the reverse effect of ICA on LDH was dose-dependent (Fig. 2b). The effects of ICA on pyroptosis in rat chondrocytes were further analyzed by detecting the expression of IL-1β and IL-18. As shown in Fig. 2c, d, LPS-induced IL-1β and IL-18 expression was reversed by ICA, and this suppression function of ICA was dose-dependent. Consistent with the qRT-PCR result, the release level of IL-1β and IL-18 protein was also decreased upon the application of ICA (Fig. 2e, f), and the suppression of IL-1β and IL-18 release level was shown to be ICA concentration-dependent. The expression of collagen formation and pyroptosis-related molecules, including MMP-1, MMP13, collagen II, IL-1β, and IL-18, were analyzed by Western blotting. Data showed that ICA dose-dependently suppressed LPS-induced promotion of MMP-1, MMP13, IL-1β, and IL-18, while prevented LPS-reduced collagen II expression (Fig. 2g, h). Taken together, these results suggested that ICA may suppress LPS-induced chondrocytes injury and pyroptosis.

### ICA inhibits LPS-induced activation of NLRP3 inflammasome and pyroptosis-related caspase-1 signaling pathway

**Fig. 3:**
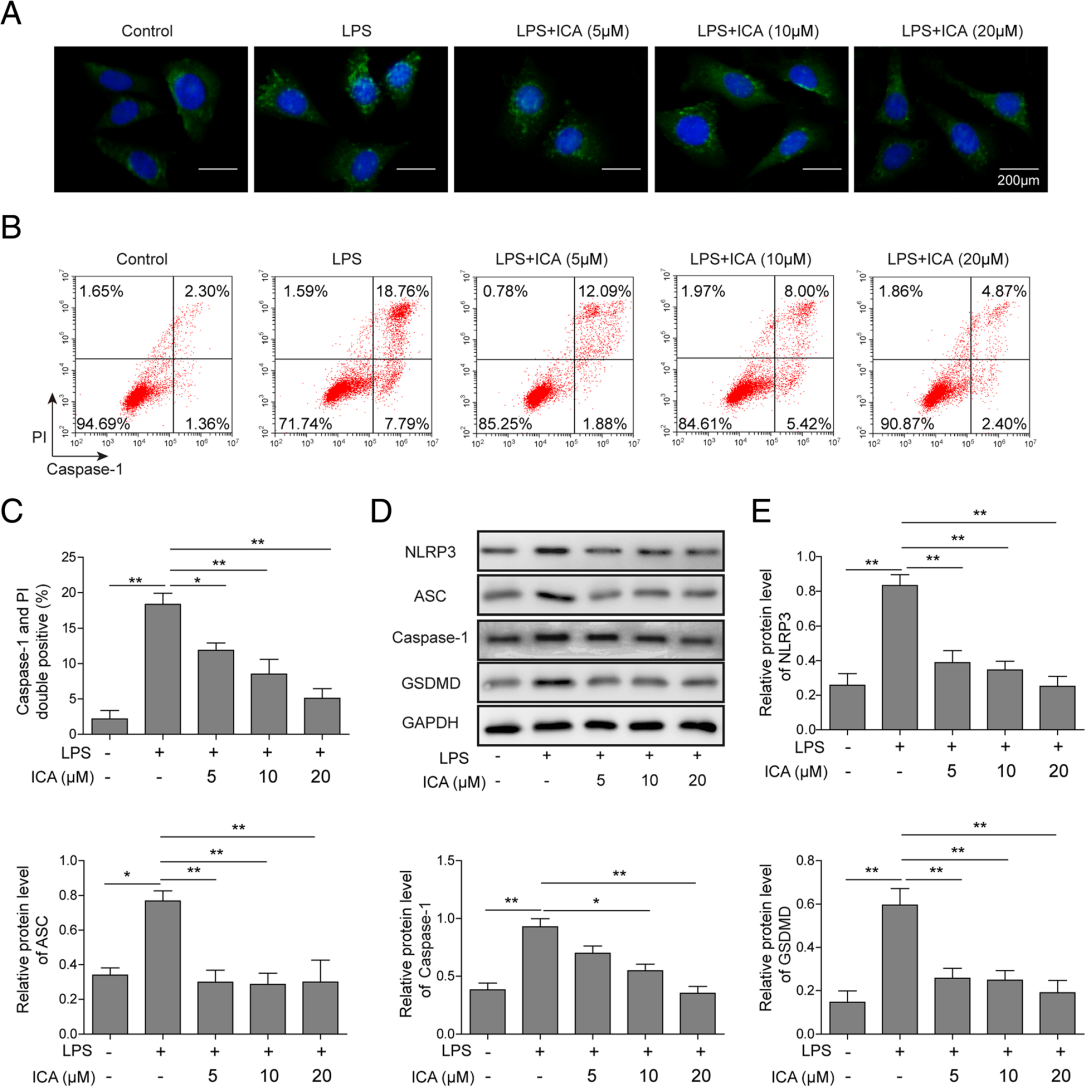
ICA exerted negative effects on LPS-induced NLRP3 expression and pyroptosis. **a** Immunofluorescent was used to detect NLRP3 in chondrocytes treated with LPS or ICA. **b** Flow cytometry analysis was used to measure caspase-1 activity in chondrocytes treated with LPS or ICA. **c** Quantitative statistics of doublepositive for caspase-1 and PI stain. **d** The protein level of pyroptosis-related protein was analyzed by Western blotting in chondrocytes treated with LPS or ICA. **e** Quantitative analysis of protein band gray in **d**. GAPDH was used as internal control. All the results were shown as mean ± SD (*n* = 3), which were three separate experiments performed in triplicate. *p < 0.05 and **p < 0.01

To further investigate the effect of ICA on LPS regulated NLRP3 expression, immunofluorescence microscopy was performed. The results demonstrated that LPS increased the expression of NLRP3 in rat chondrocytes (Fig. 3a). It was also found ICA disrupted LPS increased NLRP3 expression dose-dependently. Meanwhile, the chondrocytes pyroptosis was evaluated by flow cytometry by measuring the activity of caspase-1. As shown in Fig. 3b, c, the activity of caspase-1 was increased by LPS in chondrocytes when compared with the control group. However, the application of ICA suppressed caspase-1 activity in a dose-dependent manner (Fig. 3b, c). The levels of NLRP3, ASC, caspase-1, and GSDMD were evaluated by Western blotting, and the results revealed that ICA negatively regulated these pyroptosis-related proteins expression induced by LPS (Fig. 3d, e). These results indicated that ICA can reverse LPS-activated NLRP3 and caspase-1 signaling pathway, thus suppress LPS-induced pyroptosis in chondrocytes.

### ICA suppresses pyroptosis of rat chondrocytes through inhibitingNLRP3 signaling

**Fig. 4:**
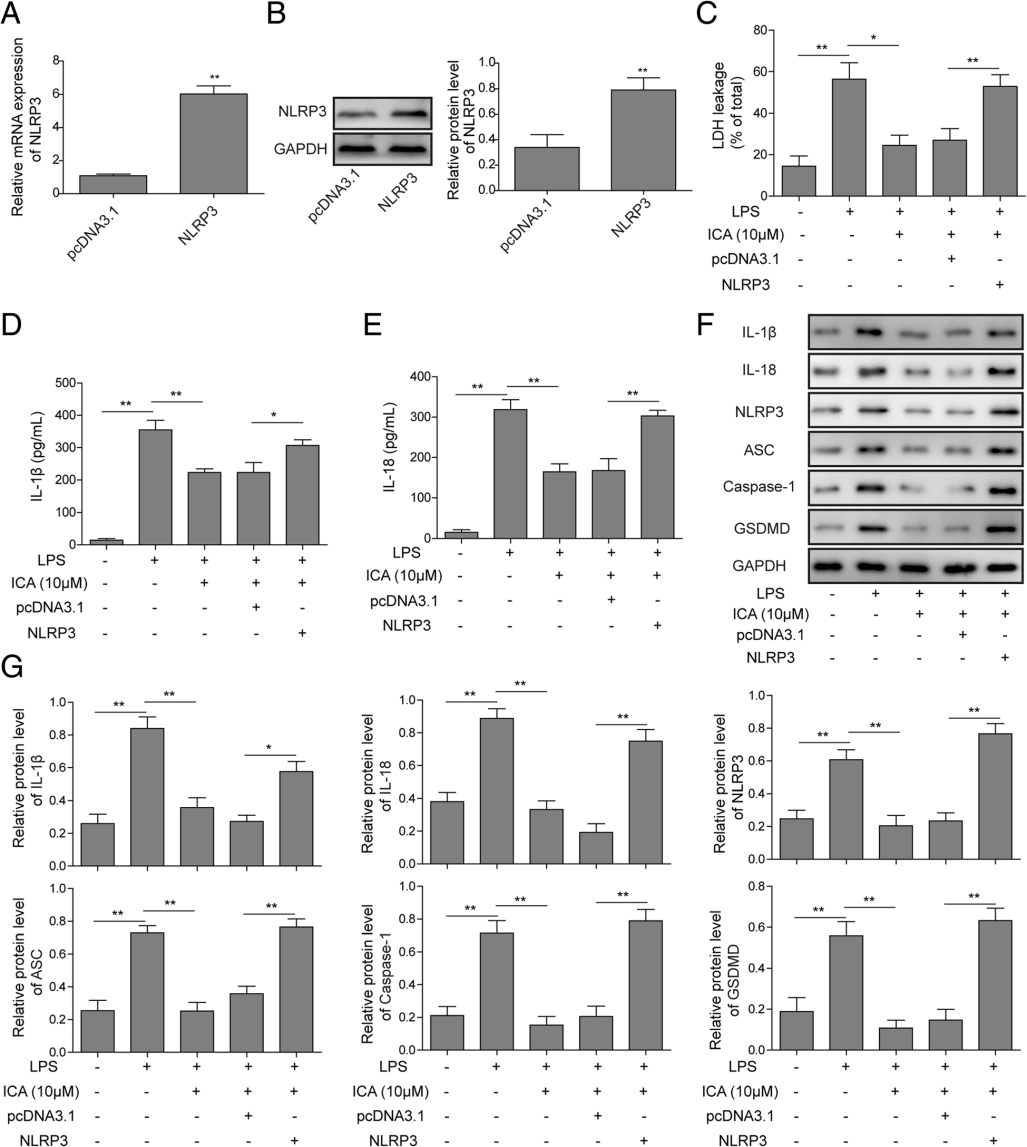
ICA suppressed chondrocytes injury and pyroptosis through NLRP3. **a** The mRNA level of NLRP3 was validated by qRT-PCR in chondrocytes. **b** The protein level was assessed by Western blotting in chondrocytes. GAPDH was used as internal control. **c** Leakage of LDH was assessed by ELISA assay in chondrocytes. The release level of IL-1β (**d**) and IL-18 (**e**) were analyzed by ELISA assay in chondrocytes. **f** The protein level of pyroptosis-related protein was analyzed by Western blotting in rat chondrocytes. **g** Quantitative analysis of protein band gray in **e**.GAPDH was used as internal control. All the results were shown as mean ± SD (*n* = 3), which were three separate experiments performed in triplicate. *p < 0.05 and **p < 0.01

To investigate the regulatory mechanism of ICA in rat chondrocytes pyroptosis, NLRP3 overexpression was constructed. As shown in Additional file 1: Figure S1, the green fluorescence intensity of the transfection efficiency increased gradually over time after transfection of the NLRP3 overexpression vector. And the green fluorescence intensity was the strongest after transfection for 48 h; thus, the transfection time was 48 h for subsequent experiments. The expression of NLRP3 was verified by qRT-PCR and Western blotting and dramatically increased NLPR3 mRNA and protein level was detected in pcDNA3.1-NLRP3 transfected chondrocytes, suggesting that NLPR3 was successfully overexpressed (Fig. 4a, b). The LDH leakage, IL-1β, and IL-18 level in chondrocytes were monitored. Consistent with former results, the LDH level and the expression levels of IL-1β and IL-18 were induced by LPS, while the application of ICA suppressed this change (Fig. 4c–e). However, overexpression of NLRP3 could reverse the effects of ICA on LDH, IL-1β, and IL-18 release levels (Fig. 4c–e). The levels of IL-1β, IL-18, NLRP3, ASC, caspase-1, and GSDMD were evaluated by Western blotting, and the results confirmed that overexpression of NLRP3 attenuated the protection effects of ICA on chondrocyte pyroptosis (Fig. 4f, g). Taken together, these results suggested that ICA-mediated suppression of chondrocytes pyroptosis is exerted by regulating NLRP3.

### ICA alleviates rat OA by inhibiting NLRP3

**Fig. 5:**
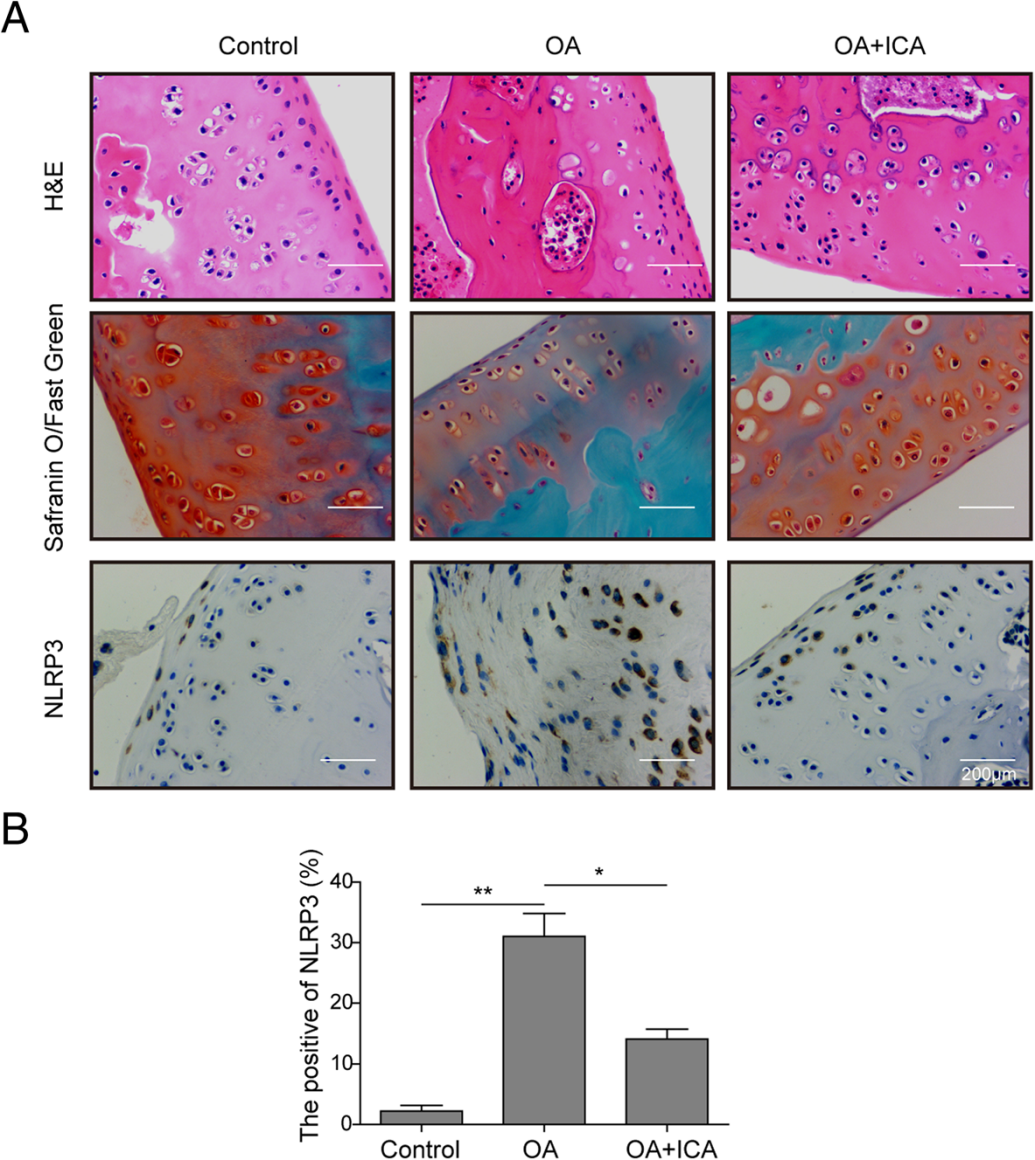
ICA protected rat against osteoarthritis. **a** H&E staining and safranin O/fast green staining were used to evaluate cartilage histopathology in OA rats, and immunohitochemical analysis was used to detect NLRP3 in OA rat cartilage tissue. **b** Statistical analysis of NLRP3 positive rate. All the results were shown as mean ± SD (*n* = 3), which were three separate experiments performed in triplicate. *p < 0.05 and **p < 0.01

To investigate the potential protective effects of ICA on osteoarthritis in vivo, we constructed a ratOA model by injecting MIA. The results of H&E and safranin O/fast green staining showed that in rat OA model, there was a severe total erosion of cartilage compared to the control group (Fig. 5a). In contrast, ICA-treated rats showed less severe destructions, which was revealed by a reduced loss of safranin O staining and surfaced regularity (Fig. 5a). The expression level of NLRP3 was detected using IHC staining. As shown in Fig. 5a, b, the rat OA model exhibited a significant increase in the number of NLRP3-positive cells compared to the control group, while the application of ICA dramatically reduced positive cell number in the rat cartilage tissue. These results clearly demonstrated that ICA is able to modulate the progression of OA and suppress NLRP3.

### ICA attenuates NLRP3-mediated pyroptosis and increases collagen formation in rat OA models

**Fig. 6:**
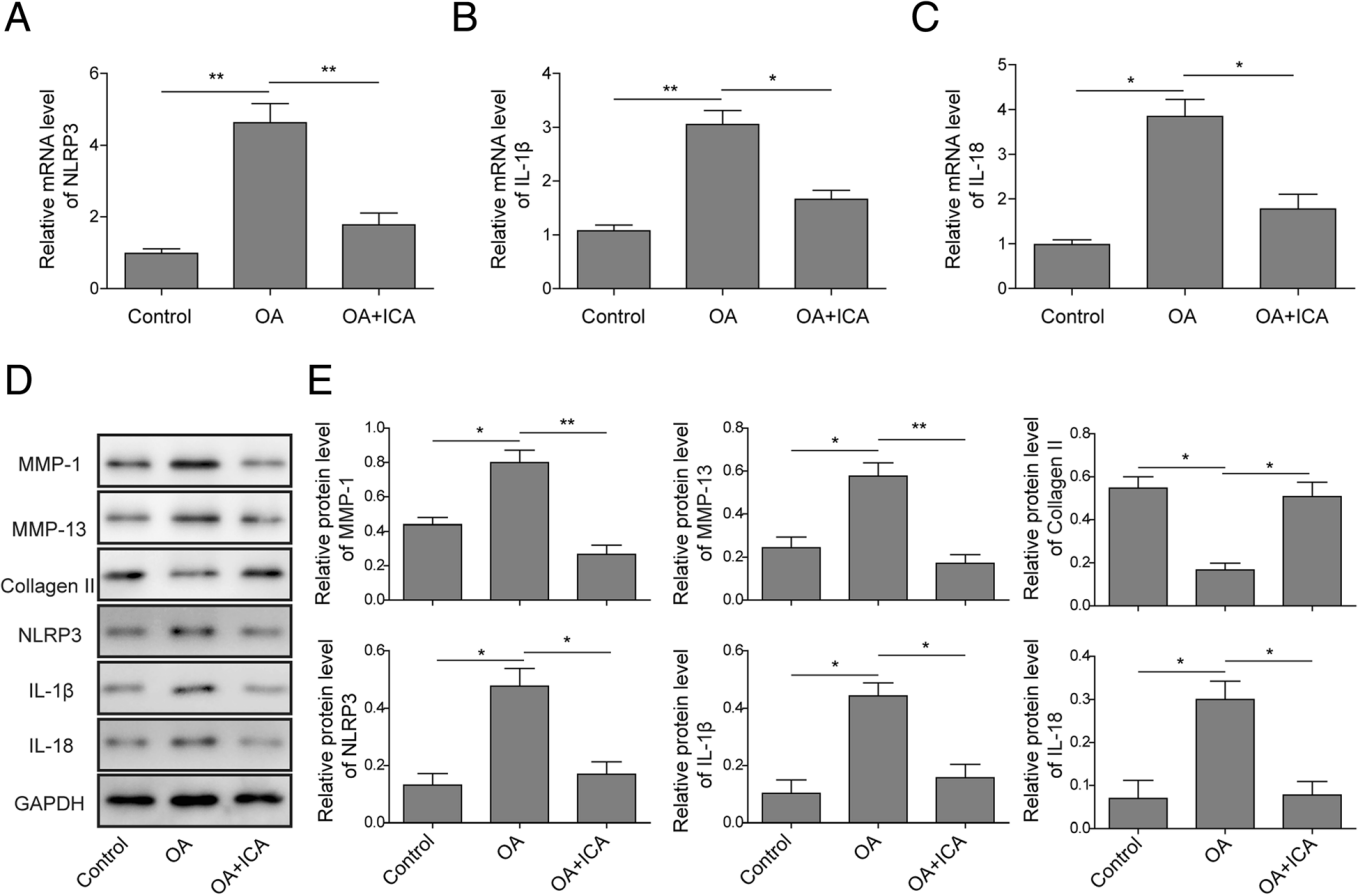
ICA attenuated pyroptosis and promoted collagen formation in OA rats. The mRNA levels of NLRP3 (**a**), IL-1β (**b**), and IL-18 (**c**) were examined with qRT-PCR in OA rat cartilage tissue. **d** The expression level of collagen formation and pyroptosis-related protein was analyzed by Western blotting in OA rat cartilage tissue. **e** Quantitative analysis of protein band gray in **d**. GAPDH was used as internal control. All the results were shown as mean ± SD (*n* = 3), which were three separate experiments performed in triplicate. *p < 0.05 and **p < 0.01

To clarify the mechanism of ICA on osteoarthritis progress, qRT-PCR was used to determine the expression of pyroptosis-related OA marker genes in rat OA models. The results showed that ICA significantly reduced the expression of NLRP3, IL-1β, and IL-18 in OA rats (Fig. 6a–c), which were consistent with the results of in vitro chondrocytes experiments. To further validate the above results, we also assessed the effects of ICA on the accumulation of pyroptosis-related OA marker proteins by Western blotting. The results revealed that the protein level of MMP-1, MMP-13, NRLP3, IL-1β, and IL-18 were suppressed while the protein level of collagen II was increased by ICA treatment (Fig. 6d, e). Collectively, these results indicated that ICA exerts a protective effect on OA by regulating pyroptosis reactions.

## Discussion

OA is a progressive and deteriorative disorder of articular joint, and current medicines including acetaminophen, nonsteroidal anti-inflammatory drugs, and duloxetine were only able to relieve the pain and unable to cure the disease. Inflammation and apoptosis are associated with the progress of OA, and anti-inflammatory agents could prevent OA development [18–21]. ICA, the extract of *Epimedium*, is known to have anti-oxidative, anti-neuroinflammatory, and anti-apoptotic effects and considered to be effective for a range of disorders, such as neoplasm, Alzheimer’s disease, cerebral ischemia, depression, diabetes, and Parkinson’s disease (PD) [22–25]. For OA, ICA was identified as an anti-osteoporotic and anti-inflammatory compound through multi-target mechanisms, including the suppression of NF-κB signaling and inhibition of MMP1, MMP3, and MMP13 expression or OPG-RANKL-RANK system via MAPK pathways [13–15]. The present study demonstrated that ICA inhibited LPS-mediated chondrocytes injury and pyroptosis and alleviated rat osteoarthritis by inhibiting NLRP3 and caspase-1 signaling. Thus, we uncovered a novel mechanism by which ICA could prevent OA, further indicating the protective effect of ICA in OA treatment.

Pathologically, MAPKs/NF-κB-mediated inflammatory response and cell apoptosis is critically involved in the development of OA. NLRP3 inflammasome has been suggested to associate with the pathogenesis of various arthritic disorders by stimulating proinflammatory cytokines and degradative enzymes [26]. A recent study reported the essential role of NLRP1 and NLRP3 inflammasomes in the fibroblast-like synoviocytes inflammation and pyroptosis, suggesting NLRP1 and NLPR3 inflammasomes could be pathologically important for OA [9]. NLRP3 may serve as a potential biomarker for the management of OA [26]. Inhibition of NLRP3 inflammasome by curcumin or estradiol could downregulate inflammatory cytokines and prevent OA progress [27, 28]. In the present study, ICA attenuated LPS-induced inflammation and pyroptosis by inhibiting NLRP3 inflammasome signaling, and the overexpression of NLRP3 reduced the protective effect of ICA. These results fully confirmed the pathological effect of NLRP3 inflammasome in OA, and NLRP3 inhibition could achieve a therapeutic effect on OA treatment.

Pyroptosis is a caspase-1-dependent type of programmed cell death activation evoked by inflammasomes, leading to cellular lysis and cytosolic contents release to the extracellular environment [29]. Gasdermin D (GSDMD), a member of Gasdermin (GSDM) family, can be cleaved by caspase-1, liberating the N-terminal domain, which oligomerizes on the plasma membrane forms a pore for the releasing of substrates, such as IL-1β and IL-18 [30]. Caspase-1-mediated pyroptosis was shown to play a role in the regulation of various diseases, such as multiple sclerosis [31], retinitis [32], and neurological disorders[33, 34]. Furthermore, caspase-1-regulated pyroptosis could control *Brucella* joint infection [35], and the absence of caspase-1 reduced joint pathology in chronic arthritis [36]. The present study further demonstrated that LPS stimulated the expression of NLRP3, promoted the activity of caspase-1, and increased pyroptosis in chondrocytes, highlighting the pathological effect of caspase-1-mediated pyroptosis in OA. ICA could interfere with LPS-mediated NLRP3/caspase-1 signaling pathway to relief OA.

## Conclusion

In conclusion, inflammasome NLRP3 plays a key role in the pathogenesis of OA. ICA could alleviate pyroptosis by inhibiting NLRP3 signaling-mediated caspase-1 pathway, thereby attenuating the damage of chondrocytes and the occurrence of OA in rats. ICA may be a promising target drug for the treatment of OA

## Additional file

Additional files 1: **Figure S1.** The green fluorescence intensity of the transfection efficiency increased gradually over time after transfection of the NLRP3 overexpression vector. (TIF 1420 kb)

## Data Availability

All data generated or analyzed during this study are included in this published article [and its supplementary information files].
