# Supplementary figures and images for: Icariin alleviates osteoarthritis by inhibiting NLRP3-mediated pyroptosis
Source: J Orthop Surg Res. 2019 Sep 11;14:307. doi: 10.1186/s13018-019-1307-6 (PMC6737611; doi:10.1186/s13018-019-1307-6)

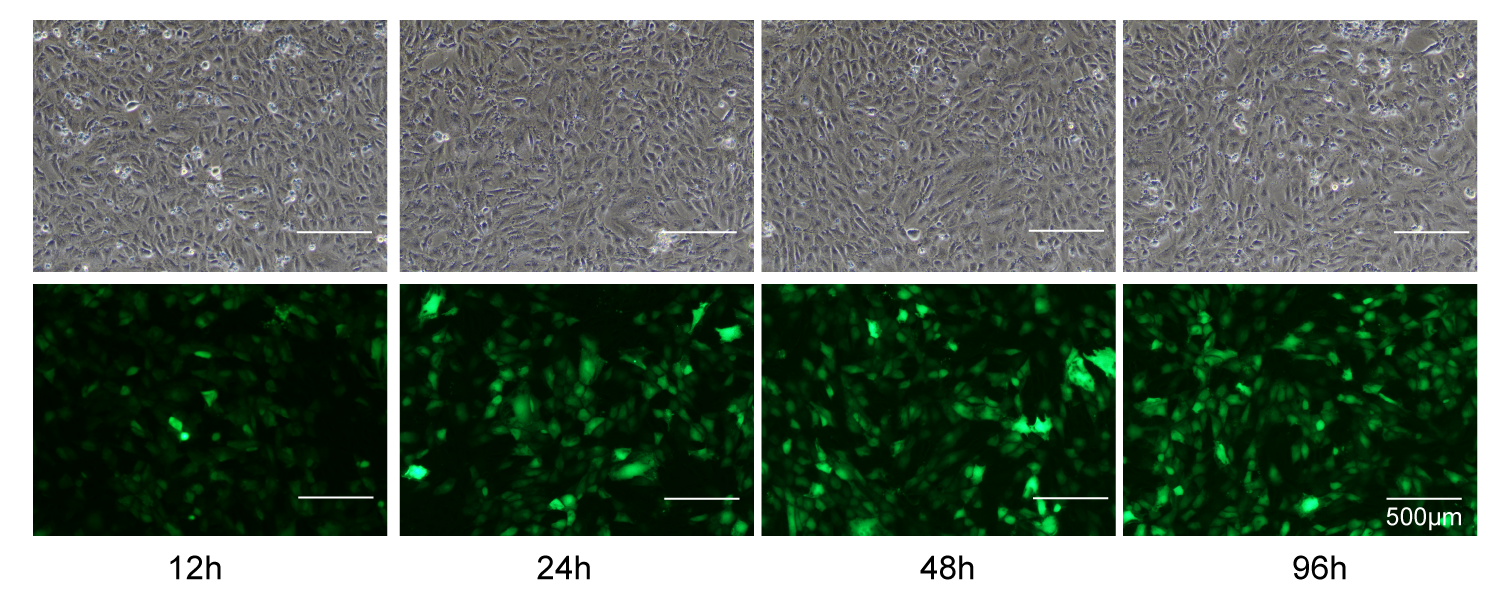

Supplement: Supplementary file 1 — Figure S1. The green fluorescence intensity of the transfection efficiency increased gradually over time after transfection of the NLRP3 overexpression vector. (TIF 1420 kb) [file 13018_2019_1307_MOESM1_ESM.tif]
